# Is Routine Group and Save Sampling Necessary for Emergency Laparoscopic Appendicectomy?

**DOI:** 10.7759/cureus.78086

**Published:** 2025-01-27

**Authors:** Simeon Harrow, Aaruran Nadarajasundaram

**Affiliations:** 1 Surgery, Maidstone and Tunbridge Wells NHS Trust, Kent, GBR; 2 General Surgery, Croydon University Hospital, London, GBR; 3 Emergency Department, St Thomas' Hospital, London, GBR

**Keywords:** emergency laparoscopic appendicectomy, general surgery, group and save, guideline, preoperative

## Abstract

Background

Laparoscopic appendicectomy is a commonly used approach for the surgical management of acute appendicitis. If complications arise, a blood transfusion may be necessary for patients undergoing emergency appendicectomy. The need for routine group and save (G&S) sampling prior to emergency laparoscopic appendicectomy remains a subject of ongoing discussion. The aim of this study was to evaluate whether routine G&S sampling is needed prior to emergency laparoscopic appendicectomy.

Methods

The present study retrospectively reviewed G&S sampling for emergency laparoscopic appendicectomy cases over a six-month period at two hospital sites in the United Kingdom: Croydon University Hospital (June 1, 2024, to November 30, 2024) and Tunbridge Wells Hospital (October 1, 2023, to April 30, 2024). A total of 304 patients across both sites were included in the review.

Results

In 2023, 23 procedures (7.6%) were performed, while 279 procedures (92.4%) occurred in 2024. The patient population consisted of 46.4% males (*n = 141*) and 53.6% females (*n = 163*), with a mean age of 37.1 years (median 33, range 6-84). A total of 406 G&S samples were collected, of which 351 (86.5%) were processed and 55 (13.5%) were rejected by the blood bank. From the sample size, only one patient received a blood transfusion prior to surgery due to a low hemoglobin level of 72 g/L, likely resulting from the delayed presentation of a perforated appendix.

Conclusion

This study suggests that omitting routine G&S sampling is safe for patients undergoing emergency laparoscopic appendicectomy. Hence, an individualized risk assessment approach should be used to identify high-risk patients requiring preoperative G&S sampling.

## Introduction

Laparoscopic appendicectomy is a commonly used approach for the surgical management of acute appendicitis, offering significant advantages including reduced postoperative pain, shorter hospital stays, and faster recovery compared to open surgery [1-3]. In patients undergoing emergency laparoscopic appendicectomy, a blood transfusion may be necessary if complications such as hemorrhage arise or if anemia is identified perioperatively. The group and save (G&S) blood test is essential for ensuring safe blood transfusion by determining the patient's blood group, providing a matched blood supply, and minimizing the risk of transfusion reactions [4].

The role of preoperative blood tests, such as G&S, in patients undergoing emergency laparoscopic appendicectomy remains a subject of ongoing discussions in surgical practice. The National Institute for Health and Care Excellence (NICE) guidelines recommend that preoperative testing should be tailored to individual risk factors [5]. In emergency laparoscopic appendicectomy, G&S tests are often performed routinely, typically involving blood typing and screening for antibodies. These tests are ordered in anticipation of potential blood transfusions due to unexpected intraoperative blood loss or complications. However, the low incidence of blood transfusion during laparoscopic appendicectomy raises questions about the necessity of routine G&S sampling for all patients [6-9].

In this study, we retrospectively reviewed G&S sampling for emergency laparoscopic appendicectomy cases over a six-month period at two hospital sites in the United Kingdom. Currently, G&S samples are collected routinely as part of preoperative assessment in both hospitals. We aim to evaluate the necessity and cost-effectiveness of routine G&S sampling in our current practice.

## Materials and methods

This retrospective analysis utilized data from electronic patient records (EPRs) at Croydon University Hospital NHS Trust (via Cerner Millennium EPR) and Maidstone and Tunbridge Wells NHS Trust (via Sunrise EPR), covering a six-month period: June 1, 2024, to November 30, 2024, and October 1, 2023, to April 30, 2024, respectively. The inclusion criteria were patients who underwent diagnostic laparoscopy and/or emergency laparoscopic appendicectomy. Patients who underwent joint surgical intervention with other specialties were excluded from this study.

After identifying eligible patients, their records were reviewed for the following: (1) number of G&S samples collected preoperatively, (2) number of G&S samples processed or rejected by the laboratory, and (3) reasons for sample rejection.

Following data collection, preoperative, operative, and postoperative notes were reviewed to assess the rate of blood transfusion in the study population.

All patient data were anonymized for this study. No identifying information was retained by the authors or included in this article. Ethical approval was granted by the local audit committee, and informed consent was not required for this retrospective study.

## Results

A total of 304 patients underwent diagnostic laparoscopy and/or laparoscopic appendicectomy across the two trusts during the specified time periods. At Croydon University NHS Trust, 118 procedures were performed, while 186 were carried out at Maidstone and Tunbridge NHS Trust. Of these, 23 (7.6%) were conducted in 2023 and 279 (92.4%) in 2024.

**Figure 1:**
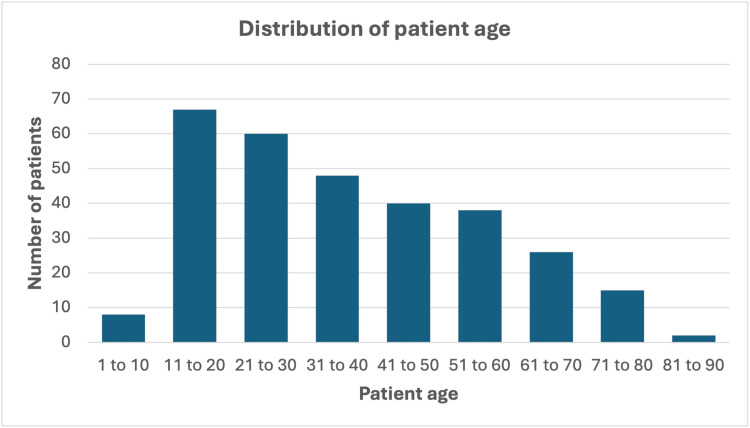
Graphical representation of patient age distribution

The patient population consisted of 46.4% males (n = 141) and 53.6% females (n = 163), with a mean age of 37.1 years (median 33, range 6-84). Age distribution is shown in Figure 1.

**Figure 2:**
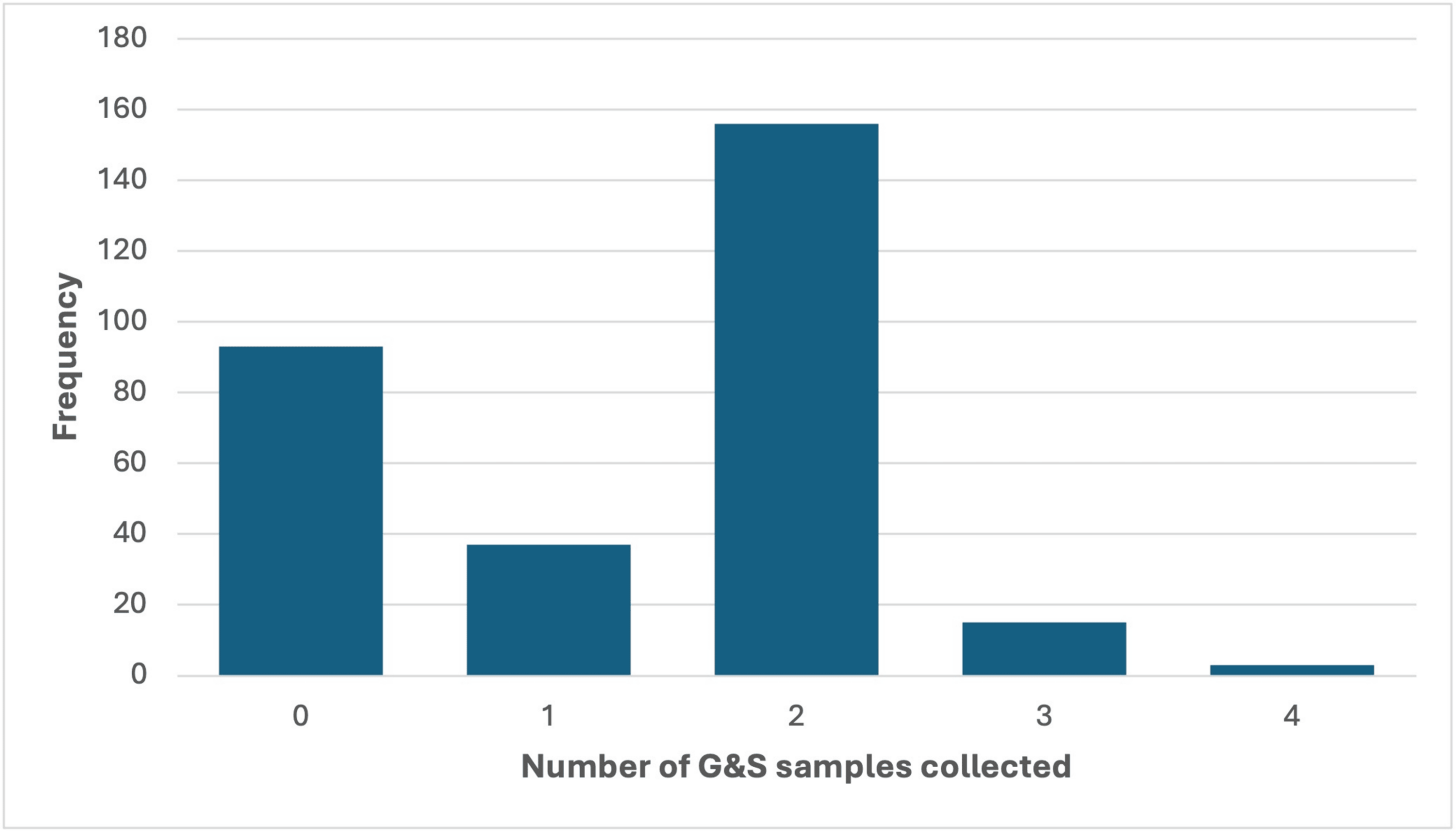
Graphical representation of the number of G&S samples collected

A total of 406 G&S samples were collected from 211 patients, of which 351 (86.5%) were processed and 55 (13.5%) were rejected by the blood bank. Of the 304 patients, 93 (30.6%) had no G&S samples collected, 37 (12.2%) had one sample, 156 (51.3%) had two samples, 15 (4.9%) had three samples, and 3 (1.0%) had four samples (Figure 2). The reasons for the rejection of the 55 samples are outlined in Table 1.

**Table 1:** Summary of reasons for sample rejection with corresponding frequencies

Reason for sample rejection	Frequency
Missing/incorrect name	4
Missing/incorrect date of birth	7
Missing/incorrect hospital number	9
Duplicate request	10
Illegible details	1
Hemolyzed	5
Not dated	4
Not signed	2
No request form received	1
Confirmatory sample taken by the same person	1
No specimen received	1

Of the 304 patients, only one patient received a blood transfusion prior to surgery due to a low hemoglobin level of 72 g/L, likely resulting from the delayed presentation of a perforated appendix, as noted in the clinical documentation.

## Discussion

The rationale for routine preoperative G&S sampling in the United Kingdom is to ensure the availability of appropriately screened and cross-matched blood products in the event of major hemorrhage. The 2016 NICE guidelines for routine preoperative tests in elective surgery recommend testing based on patient comorbidities; however, G&S sampling is not included in these assessments [10]. At present, there is no national guidance on the use of routine G&S sampling in emergency laparoscopic procedures [4].

Safety

Laparoscopic surgeries are widely recognized for their safety, particularly concerning the minimal need for perioperative blood transfusions. Several studies have documented exceptionally low transfusion rates associated with these procedures. White and Armstrong evaluated the necessity of routine preoperative G&S sampling in laparoscopic appendicectomy and reported a zero incidence of blood transfusion, suggesting that routine G&S sampling may not be necessary in low-risk patients [11].

Additionally, a retrospective study by Ramasamy et al. assessed the use of preoperative G&S samples in laparoscopic surgeries, including appendicectomies. The study reported no patients required perioperative transfusions, suggesting that routine G&S sampling is not a cost-effective practice [12].

These studies collectively highlight the safety of laparoscopic appendicectomy and the low transfusion rates associated with the procedure, supporting the need to review the routine use of preoperative G&S sampling in all patients.

Risk assessment

Implementing individualized risk assessments for potential blood transfusions in laparoscopic procedures is of utmost importance for optimizing patient care and resource utilization. Adopting an individualized risk assessment approach for G&S sampling could lead to a significant reduction in costs and laboratory workload, without compromising patient safety. Individualized risk assessment could consider factors such as preexisting comorbidities, previous abdominal surgeries, current hemoglobin level, and coagulation studies. A study by Ghirardo et al. noted that patients who received perioperative blood transfusion had an existing condition, rather than a direct consequence of the surgery [13]. This coincides with the findings of our study, whereby only one patient required a blood transfusion due to their suboptimal hemoglobin level preoperatively.

In addition, a recent study by Chaudhari et al. evaluated the need for routine G&S sampling prior to emergency laparoscopic appendicectomy [14]. They concluded that selective sampling based on individual risk factors is more appropriate, emphasizing this approach can optimize resource utilization in healthcare settings [14]. Therefore, by adopting a tailored approach to G&S sampling, healthcare providers in the United Kingdom can significantly improve efficiency while maintaining a high standard of patient care.

Furthermore, a machine learning model developed by Lou et al. demonstrates that incorporating both surgery-specific and patient-specific variables enhances the prediction of transfusion risk, resulting in improved allocation of blood bank resources and anesthetic planning [15].

Cost-effectiveness and work efficiency

A systematic review by Fadel et al. highlighted the potential cost savings that could be made through selective G&S sampling, where sample costs range from £15.00 to £21.30 [16]. Consequently, the calculation of the total number of processed G&S samples from our institutions, using the previously mentioned cost range, totals £5,265-£7,476.30 over the measured time periods.

In addition, we note from our study that the most common reason for sample rejection was duplicate requests, further increasing clinical workload as well as unnecessary blood tests for patients. This highlights further inefficiencies in current practice, whereby unnecessary samples are often collected due to the potential difficulties with confirming valid G&S status. This shows further teaching is required to raise awareness regarding the confirmation of G&S status and when additional samples may be required.

Furthermore, two staff members are required to collect one G&S sample each, at least 30 minutes apart, for a valid result. This is often challenging due to work pressures and reduced staffing during off-hours.

Limitations

Throughout this study, we have noted several limitations. We have suggested omitting routine G&S sampling is a safe practice and proposed G&S samples should be collected following an individual risk assessment for high-risk patients. However, we were not able to clearly define criteria for patients deemed high risk. Furthermore, when reviewing the reasons for sample rejection listed in Table 1, we were not able to confirm the contributing factors, likely due to human error. While we acknowledge our study reviewed patient data sets from two independent NHS Trusts, the time period over which data was collected was limited. Data was collected over a six-month period; however, a longer study duration, as well as involving additional NHS trusts, could provide further insight to identify high-risk patients. This could provide further scope for establishing G&S sampling guidelines for emergency laparoscopic appendicectomy. 

## Conclusions

Our study suggests that omitting routine G&S sampling is safe for patients undergoing emergency laparoscopic appendicectomy. Adopting an individualized risk assessment approach would help identify high-risk patients who require preoperative G&S sampling. This could significantly reduce unnecessary blood tests, improving clinical workload and alleviating financial burden without compromising patient care.
